# 

**Published:** 1870-10

**Authors:** 


### ARTICLE IV.

### *Virginia State Dental Society.*

*Editor American Journal of Dental Science.*—Dear Sir: I am requested to announce through the pages of your journal, that a meeting of the Dentists of the State of Virginia, is proposed to be held in Richmond, on the third day of November next, for the purpose of organizing a "State Dental Society" and for the transaction of such other business as may come before the meeting.

It is proposed to imitate the example lately set by the Medical Faculty of the State, in memorializing the Legislature to secure the passage of an act for the better protection of the regular practitioners of dentistry in this State, and thus aid in elevating the standard of the profession.

Dentists of this State and others are invited to attend. Any further information may be obtained by addressing Dr. James F. Thompson, of Fredericksburg, Va., or the undersigned James B. Hodgkin, Alexandria, Va.

### ARTICLE V.

OFFICE CORRESPONDING SECRETARY,
SOUTH CAROLINA STATE DENTAL ASSOCIATION,
COLUMBIA, S. C. Sept. 27th, 1870.

*To the Editor of the American Journal of Dental Science:*

DEAR SIR:—You will greatly oblige the members of the South Carolina State Dental Association, by inserting the following in your very valuable and widely circulated journal:

"SOUTH CAROLINA STATE DENTAL ASSOCIATION."—The regular semi annual meeting of this association will be held in Charleston, S. C., commencing Tuesday Nov. 1st, at 8 o'clock, P. M. All dentists in the State are cordially invited to attend, and brother dentists from other States will receive a hearty welcome.

The usual Railroad reductions of going and returning for one fare will be extended to members, &c.

THOMAS T. MOORE, *Cor. Sec.*

---

### SELECTED ARTICLES.

---

